# Social influence on 5-year survival in a longitudinal chemotherapy ward co-presence network

**DOI:** 10.1017/nws.2017.16

**Published:** 2017-07-12

**Authors:** JEFFREY LIENERT, CHRISTOPHER STEVEN MARCUM, JOHN FINNEY, FELIX REED-TSOCHAS, LAURA KOEHLY

**Affiliations:** National Human Genome Research Institute, NIH, Bethesda, MD, USA and CABDyN Complexity Centre, Saïd Business School, University of Oxford, Oxford, UK; National Human Genome Research Institute, NIH, Bethesda, MD, USA; Oxford University Hospitals, National Health Service, Oxford, UK; CABDyN Complexity Centre, Saïid Business School, University of Oxford, Oxford, UK and The Oxford Martin School, University of Oxford, Oxford, UK; National Human Genome Research Institute, NIH, Bethesda, MD, USA

**Keywords:** medicine, public health, chemotherapy, longitudinal network, communal coping, generalized estimating equation, jaccard index, administrative data

## Abstract

Chemotherapy is often administered in openly designed hospital wards, where the possibility of patient–patient social influence on health exists. Previous research found that social relationships influence cancer patient’s health; however, we have yet to understand social influence among patients receiving chemotherapy in the hospital. We investigate the influence of co-presence in a chemotherapy ward. We use data on 4,691 cancer patients undergoing chemotherapy in Oxfordshire, United Kingdom who average 59.8 years of age, and 44% are Male. We construct a network of patients where edges exist when patients are co-present in the ward, weighted by both patients’ time in the ward. Social influence is based on total weighted co-presence with focal patients’ immediate neighbors, considering neighbors’ 5-year mortality. Generalized estimating equations evaluated the effect of neighbors’ 5-year mortality on focal patient’s 5-year mortality. Each 1,000-unit increase in weighted co-presence with a patient who dies within 5 years increases a patient’s mortality odds by 42% (*β* = 0.357, CI:0.204,0.510). Each 1,000-unit increase in co-presence with a patient surviving 5 years reduces a patient’s odds of dying by 30% (*β* = −0.344, CI:−0.538,0.149). Our results suggest that social influence occurs in chemotherapy wards, and thus may need to be considered in chemotherapy delivery.

## 1 Introduction

Cancer is a leading cause of death in the United Kingdom, with one in four people dying of cancer (UK Cancer Research, 2014). Cancer patient outcomes, particularly the gold-standard 5-year survival, have been robustly associated with a number of individual characteristics such as treatment protocol, age, sex, and cancer severity (Bossard et al., 2007; Coleman et al., 2011; Siegel et al., 2015). A patient’s social sphere may also impact patient outcomes: there is evidence, for example, that social ties (e.g., increased social network size and interactions) are associated with both reduced all-cause mortality and cancer-specific mortality, and that this relationship may be stronger for women (Ernst et al., 1992; Holt-Lunstad et al., 2010; Shor & Roelfs, 2015). However, there is limited research considering the effect of the social context of cancer treatment itself on patient survival. Indeed, chemotherapy—one of the most common forms of treatment—is often administered in outpatient group settings, representing one important social context for further inquiry. To this end, we investigate the impact of network members’ co-presence, survivorship, and death on individual chemotherapy patients’ survival.

### 1.1 Stress-mediated effect of social influence on health

Social influence may be an important interpersonal mechanism impacting health outcomes of cancer patients receiving treatment in the chemotherapy ward through patients’ stress response. Patients entering chemotherapy generally have three concomitant threats to their health: the physical disease of the cancer, the neuro-endocrine stress based on uncertainty related to the course of the physical disease, and the cellular response to chemotherapy (Maria et al., 2004; Tiligada, 2006). Although the cancer itself is the major cause of mortality, the effects of stress on health are also important; reduced stress can significantly reduce 5-year mortality in chemotherapy patients (Cohen, 1988). Therefore, if stress is altered by some social influence process, then such processes can impact overall health and survival of cancer patients. A spectrum of social influence mechanisms exists, each distinguished by variation in the type of interaction.

Each has different implications for how social influence may impact the patient’s stress response. The strongest effects of social influence are in the context of direct interactions with close social ties (Beaton et al., 2008; Holt-Lunstad et al., 2010). This work generally considers the exchange of social resources, including informational, emotional, and instrumental support (Cobb, 1976; House, 1987). There are a number of robust associations between the structure and function of patients’ social support networks, patients’ stress response, and ultimately their health outcomes. These include the strength of ties (Orth-Gomer & Johnson, 1987; Yang et al., 2016), received social support (Luszczynska et al. 2007), and perceived social support (Yang et al., 2016). Such support resources have been shown to influence how patients with a health stressor manage that stress (Berg et al., 2008). For example, Croyle & Hunt (1991) showed that patients experienced different responses to a medical test result based on whether or not they interacted with a confederate receiving the same test result; those receiving similar results experienced lower stress levels than those that differed. However, not all social interactions result in positive outcomes. For instance, patients can also support one another via an exchange of information (Frost & Massagli, 2008), but if the information is poor, one’s perception of the sharer’s knowledge is negative, or the relationship between network members itself is negative; the health outcomes of this interaction can be deleterious (McKinlay, 1973; Rook, 1984).

Social influence may also occur in the absence of direct interaction. A patient’s physical appearance, for example, may convey information about their true health status, which other patients observe; such information can impact, in turn, the observer’s health. Previous research has shown that the mere presence of others engaged in the same task impacts physiological arousal and performance, in what is known as social facilitation (Zajonc, 1965). Also, individuals will alter their behavior based on the behavior of those around them, in what is known as modeling (De La Haye et al., 2011). While there is not a specific behavior or task in chemotherapy, one still observes others responding well or poorly to treatment over time. These observed responses could then effect arousal or stress pathways, resulting in indirect influence dependent on the outcomes of the observed patients. Both social facilitation and modeling can therefore result in differences in behavior and stress response that ultimately impact health.

Finally, it is important to note that these social processes may result in differential stress responses based on whether the relationship in question is with a stranger or a familiar individual. For example, one study found that people had slower latent cognitive reaction times based on functional Magnetic Resonance Imaging (fMRI) when presented with a familiar face when compared to an unfamiliar face. Such responses likely indicate increased arousal for participants when observing familiar faces (Beaton et al., 2008), a result previously found in animal models (Armario et al., 1983). Thus, observing familiar people elicits different reactions than observing strangers. In the setting of chemotherapy, patients almost always begin as strangers, but may become more familiar over time (whether through direct interaction or observation and mitigated through a process of consistently being co-present together), and those who do become familiar may exert stronger influence on one another.

### 1.2 Social influence in other social contexts for chemotherapy patients

There is strong evidence to suggest that social influence can impact stress and therefore health outcomes through a variety of interpersonal processes. Much of the research investigating social influence processes in cancer patients has involved patients in settings outside the chemotherapy ward. For example, couples support one another when one member has developed cancer. Although only one member is affected by cancer, both experience stress due to the diagnosis and both are likely to engage adaptive changes to the stress by supporting each other (Berg et al., 2008). In this context, couples have a pre-existing relationship in which members intimately know and support each other prior to the cancer diagnosis.

There is also a large body of evidence that member of cancer social support groups benefit from exchange of social support resources (Cain et al., 1986). While patients in cancer support groups likely do not know each other prior to joining the group, patients join such groups because they are seeking and in need of support from experientially similar others. Additionally, all patients entering into cancer support groups self-identify as persons with cancer, which can enhance the social interactions with other, like-minded individuals. This is particularly important, as those with similar experiences are ideally situated to influence others’ stress buffering via emotional sustenance, assistance in active coping, or role modeling (Thoits, 2011). Such support groups represent a context in which patients form a rapport *de novo,* followed by subsequent strengthening or weakening of their relationship.

In contrast, patients within the chemotherapy ward may or may not be actively seeking social support from others receiving chemotherapy; their primary purpose is cancer treatment. Any social exchange occurring while receiving chemotherapy is secondary to treatment. However, the chemotherapy ward is an important social context to investigate whether social influence naturally occurs, and if so, what impact such influence has on patients’ health outcomes. We hypothesize that social influence can be both positive and negative. For example, we would expect that positive patient outcomes, such as cancer survivorship, would positively impact patients receiving treatment together. However, negative patient outcomes, such as physical decline and death, may negatively impact the survivorship of patients receiving treatment together. The latter proposition is based on evidence indicating that the death of a close network member, such as a spouse, likely accelerates one’s own mortality (Elwert & Christakis, 2008; Mineau et al., 2002). More generally, previous research has shown that disruption in one’s social network can adversely affect psychological distress that can lead to adverse health outcomes (Oyama et al., 2012; Perry, 2016). Noticing that familiar patients are no longer in the chemotherapy ward (due to death or successfully finishing chemotherapy) may have similar effects if one considers those others as members of their social network. Thus, the chemotherapy setting represents an ideal context to investigate the influence of patient outcomes and network disruption on survivorship and whether such influence can occur when neither pre-existing social ties nor an explicit desire for social support are present.

### 1.3 Social networks

Past research has alluded to the role of social network resources in cancer patients’ health trajectories, but has not made use of sociometric data, primarily due to the cost and difficulty associated with its procurement. These studies have predominantly focused on very small networks of couples (Oberst et al., 1989), been primarily qualitative (Wolf, 2015), or have used data from a subset of the people represented in the network (Koehly et al., 2011). However, all of these studies build the evidence base that the connections between people within a network have important implications for social influence processes that facilitate cancer patients’ adaptation to their diagnosis and treatment. Moreover, there is a breadth of research showing that social influence is impacted by network structure for a variety of health outcomes, and this is robust across age, race, and socio-economic status (Christakis & Fowler, 2007; Mercken et al., 2012; Perkins et al., 2014; Valente et al., 2003). Indeed, this body of research focuses primarily on established social relationships or support group settings and shows that influence does not depend solely on individual, independent dyads or a direct social connection, but that the overall structure of the network is important. There are methodological issues when examining influence processes within such contexts—differentiating between selection or influence is generally difficult—limiting the types of inferences one can make (Shalizi & Thomas, 2012; VanderWeele, 2011). However, the chemotherapy ward is not a context within which one self-selects or chooses partners, providing a unique opportunity to evaluate influence processes with a limited possibility for social selection bias.

### 1.4 Research questions and hypotheses

In the current paper, we investigate the influence of cancer patient health outcomes on individual 5-year survival in a longitudinal chemotherapy treatment co-presence network. We do so in the context of the chemotherapy ward, where patients are unlikely to have previous social ties with others in the ward, and who are not present primarily to seek social support. Despite this, there is ample opportunity for social influence to occur either directly via the exchange of social support resources or indirectly by consistent observation of other patients’ health changes over time. As such, health outcomes of those with whom cancer patients are consistently co-present can potentially influence individual patient outcomes. To this end, we investigate whether co-presence between chemotherapy patients is associated with survival and whether such influence mechanisms vary by sex (Shor & Roelfs, 2015).

## 2 Data and methods

Our data come from the Infections in Oxfordshire Research Database, which is composed of standard National Health Service (NHS) administrative records. This data set was originally established to monitor infectious diseases (e.g., recording full genotyping of infectious agents), and also contains complete individual health records. The data for our analysis comprises all 4,691 patients in the hospital’s single outpatient chemotherapy ward from Jan 1, 2000 to Jan 1, 2009. We exclude 49 patients who received chemotherapy for conditions unrelated to cancer (e.g., for multiple sclerosis). Our data also include 109 left-censored cases (representing 2% of cases) who were already receiving chemotherapy at the time data collection began. Each patient’s medical history is broken into individual visitation spells (*n* = 43,898) in the chemotherapy ward with time stamps for both entry and exit. A chemotherapy spell is defined as every occurrence with unique entry and exit times of any patient into the chemotherapy ward. The date of death is also recorded for each individual through June, 2015.

### 2.1 Chemotherapy ward and process of treatment

**Fig. 1:**
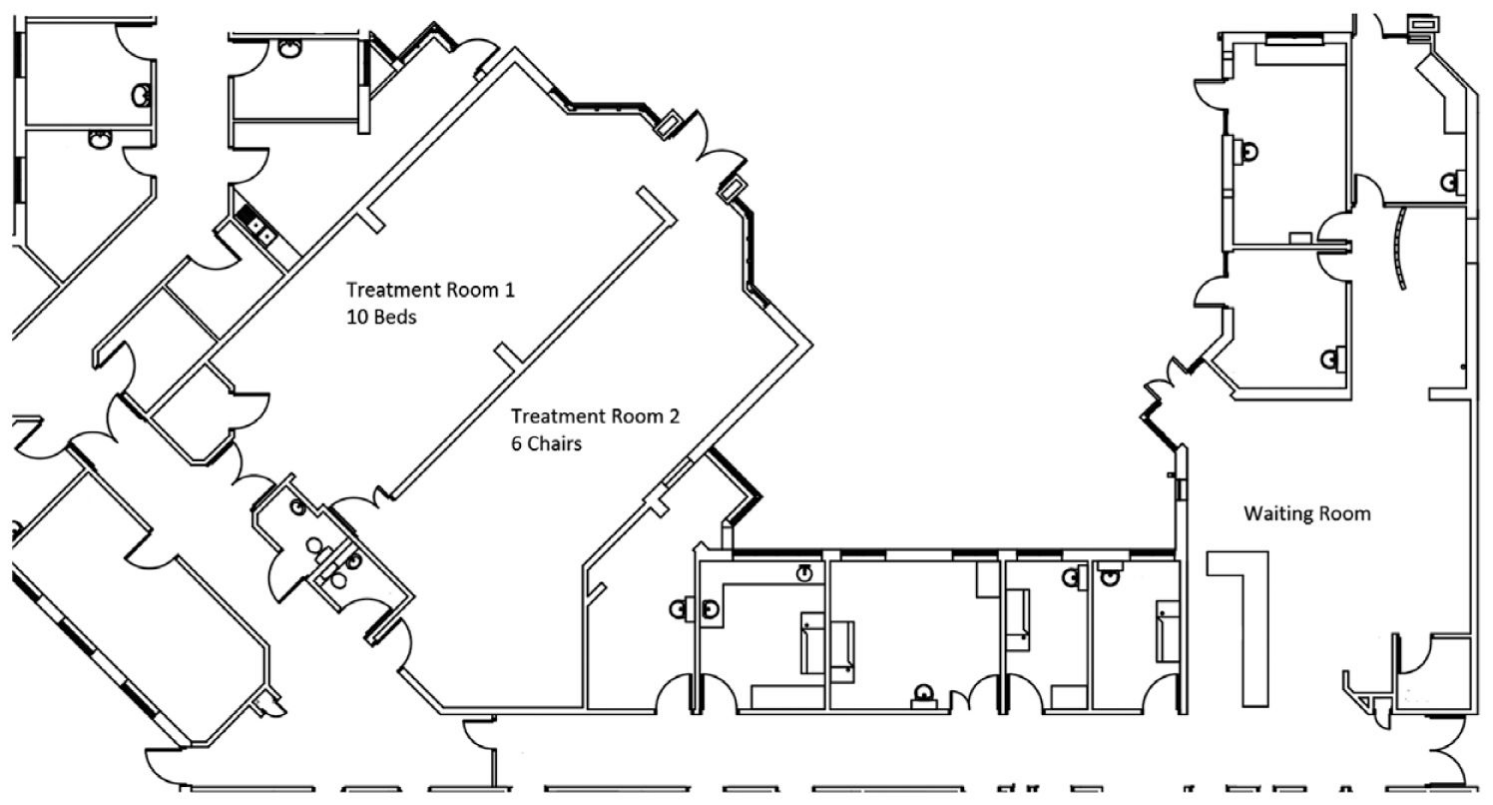
Layout of the chemotherapy ward. Patients begin spells in the waiting room, and are taken to either treatment room 1 or 2 depending on a number of factors.

As an outpatient setting, the chemotherapy ward was open between 8 a.m. and 8 p.m. from Monday through Friday. The ward was split into two treatment rooms, containing 10 beds and 6 chairs, respectively, arranged in a circle (Figure 1). The beds were fitted with a screen that could be drawn when privacy was desired. Other than that, all patients in the same treatment room were in view of one another for the duration of treatment. Upon arriving to the ward, patients began in the waiting room and underwent bloodwork to ensure eligibility for chemotherapy. This was typically done on the day of treatment, but could be done the day before. Depending on the results of the blood test, chemotherapy could commence, be postponed, or canceled.

Chemotherapy regimens are stringent in the timing of doses. The timing of a patient’s initial dose is based on the importance of immediate treatment, availability in the ward and patient preference. Once the first treatment is scheduled, the rest of the treatments follow a standard schedule, with a few hours of variation on any given day depending on patient availability. Therefore, patients with whom one overlaps are primarily determined by who is in the ward at the time of the initial dose, and the prescribed timing of chemotherapy. This informs pairs of individuals for which we consider influence possible.

### 2.2 Co-presence network construction

The network of interest represents patient co-presence in the chemotherapy ward. Because we want the connections to represent quantities of co-presence with the potential for social influence, and no measure perfectly captures all the dimensions we think are important for social influence, we use two different methods for determining co-presence. The primary network measure is the Jaccard index, the matrix of which is defined as the intersection of the two patients’ treatment times divided by the union of these patients’ treatment times. In other words

$$J_{ij}=\frac{|H\;(i)\cap H\;(j)|}{|H\;(i)\cup H\;(j)|},$$

where *H*(*i*) is a function that returns the set of hours patient *i* spent in the chemotherapy ward during the course of their treatment, and its magnitude is the total number of hours patient *i* spent in the ward. We subset the denominator based on the time patients *i* and *j* could have spent together; i.e., when they are both alive and undergoing chemotherapy. This gives a quantitative measure of how often *i* and *j* were together relative to how often they could have been together, resulting in a weighted and undirected network such that *J_ij_* = *J_ji_*_,_ and *J_ij_* = 0 indicates 0 hours of overlap between *i* and *j*.

However, the Jaccard index has some limitations. One such limitation is its lack of sensitivity to total patient chemotherapy hours; when two patients overlap and are only in the ward for a single spell, their Jaccard index is very high and indistinguishable from two patients who are repeatedly in the ward together over time. This is evident in the large peak in the empirical density of the Jaccard index at one (Figure S1). Of those, 1,762 Jaccard indices stem from single-visit overlaps. To address this, we up-weight the Jaccard index by the hours spent in the ward by the focal patient (see independent variables). As a second limitation, the Jaccard index proposes that even an hour of co-presence can result in social influence. It is our belief that this is not theoretically likely. We address this potential issue by also constructing a network based on consistent co-presence—that is, we create an edge when two patients overlap more than expected by chance, which has the benefit of defining a baseline expectation for overlap given ward scheduling. This method and the parallel analyses are presented in the Supplementary Information (see Figure S2 and Table S1, Model A).

### 2.3 Dependent variable

Our primary outcome is a patient’s 5-year mortality, which is the gold-standard in cancer survivorship research and practice (Bossard et al., 2007). Patients’ outcomes were measured at the end of treatment by recording the last chemotherapy session followed by a 6-month period with no visit to the chemotherapy ward. The outcome was recorded 1 = death if they died within 5-years of their end of treatment date and had a diagnosis of cancer at the hospital spell most temporally proximate to their date of death (i.e., count as censored patients who die of causes unrelated to their cancer), and 0 = survival otherwise. Because the chemotherapy data ends in 2009, and we have death data through mid-2015, there is no administrative censoring for 5-year survival—we treat *de facto* right-censoring after 2015 simply as 5-year survival.

### 2.4 Independent variables

#### 1-path Influence

As previously noted, complete overlap of treatment, whether over 1 or 100 hours, will result in equivalent Jaccard indices. We, therefore, weight the Jaccard index by the number of hours the focal patient spent in the ward. We call this the Jaccard-weighted person-hours, which differentiate overlap of only a few hours from that of many hours while still adjusting for the relative potential for overlap by any two patients. The Jaccard-weighted person-hours for a focal patient can be written as:

$$JW\;(i)=\;|H(i)|\sum_{j}J_{ij},\;\;\;j\neq i.$$

This total count is then partitioned into the Jaccard-weighted person-hours with directly- connected patients who survived at least 5 years following chemotherapy (JW*_S_*_1_*_P_* ) and with those who died within 5 years following chemotherapy (JW*_D_*_1_*_P_* ), allowing us to separate their relevant effects and understand more about underlying influence mechanisms. These can be written as:

$$JW_{S1P}\;(i)=|H(i)|\sum_{j}\left(J_{ij}*S(j)\right),\;\;\;j\neq i\;JW_{D1P}\;(i)=|H(i)|\sum_{j}\left(J_{ij}*(1-S(j))\right),\;\;\;j\neq i,$$

where *S*(*j*) is equal to one if patient *j* survived at least 5 years following their chemotherapy and zero otherwise.

#### 2-path Influence

Although we *a priori* expect the influence to be between directly-connected patients only, we include 2-path influence variables representing the sum of Jaccard-weighted person-hours for paths to non-adjacent patients two-steps away from the focal patient based on their outcomes (survival or death) as a negative control. While it is possible that a patient two-steps away may influence a directly-connected patient, we believe there would be no influence independent of the mediating patient, and such an effect should be non-significant. When an open 2-path exists, it means that the patients without an edge did not overlap at all, despite being in chemotherapy around the same time (as evidenced by their common overlap with a third patient). This would occur when the start and end patients just missed each other temporally in the ward, most likely reflecting random scheduling variation based on patient or ward availability. The start and end patients in the 2-path would, therefore, be in the ward together were it not for random chance. Additionally, because they both overlap with similar patients, any latent characteristics leading to co-presence would also likely be similar. Although a significant result for these covariates could be due to either latent similarity between patients two-steps away or due to influence over two steps in the network, the lack of a significant result indicates that neither of these mechanisms are likely at work *ceteris paribus.* We, therefore, construct the variables as total weight of open two-paths between a focal patient and another patient based on whether the other patient survived (JW*_S_*_2_*_P_* ) or died (JW*_D_*_2_*_P_* ). Weights of the open 2-paths were equal to the product of the two individual edge weights on the path. These can be written as:

$$JW_{S2P}\;(i)=|H(i)|\sum_{j}\sum_{k}\left(J_{ij}*J_{jk}*S(k)*M_{ik}\right),\;\;\;j\neq i,k\;JW_{D2P}\;(i)=|H(i)|\sum_{j}\sum_{k}\left(J_{ij}*J_{jk}*(1-S(k))*M_{ik}\right),\;\;\;j\neq i,k,$$

where *M_ik_* equals 1 when *i* and *k* are not directly connected in the co-presence network (i.e., they are never co-present), and zero when they are.

### 2.5 Covariates

A number of covariates are included in our fitted models as controls for other sources of heterogeneity. First, we include patient sex and the age of the patient at the start of chemotherapy. In addition, we control for variables related to the chemotherapy treatment itself. These include the number of visits to the ward during the chemotherapy cycle and the total time in the ward over all ward visits. As well, we include the timing with respect to the 10 years of observation of when each patient began their chemotherapy, allowing us to control for any exogenous changes to survival as treatment improved over time. We also control for the total person-hours of overlap each patient had with all others in the ward. Finally, we add two terms to the model for the interaction between patient sex and the direct path Jaccard-weighted person-hour statistics.

To account for the effect of disease severity on 5-year survival we include variables derived from the ICD-10 codes (C00-C99) on patients’ health records (WHO, 1992). Previous studies largely focused on a single type of cancer and adjust for the disease stage (Kroenke et al., 2006). No studies could be found that adjusted for the severity of cancer across primary cancer sites, giving us no basis from past literature for which to adjust for disease severity. Thus, we generalized past approaches by including all primary cancer types as a series of dummy variables, one for each observed cancer type, for a total of 20 variables, with pancreatic cancer as the arbitrary baseline. Empirical 5-year survival in these data ranged from 7% of patients for brain cancer to 94% for prostate cancer, indicating a large variance in basal prognosis with respect to the location of the primary cancer (UK Cancer Research, 2014). Patients that were recorded as having non-specific, ill-defined, secondary, or miscellaneous multiple sites (i.e., from ICD codes C76, C77, C78, C79, C80, C97) were given their own dummy variable as these cancers are typically more rare and more difficult to treat (Shibuya et al., 2002).

Although the ICD-10 code does not explicitly differentiate between stages of cancer, we can distinguish Stage IV, the most severe cases, where the neoplasm has aggressively spread to a second tissue with a secondary cancer diagnosis (metastasized) (AJCC, 2017). We include an indicator variable for any secondary cancer diagnoses during a patient’s treatment as a proxy for metastasis. Left-censored patients (*n* = 145) had this variable imputed based on the full-data set as a function of a patient’s covariates. Model checking diagnostics revealed that our results were robust to this imputation.

Finally, we control for the admitting consultant physician. The admitting consultant physician can induce latent homophily among patient outcomes, if their patients are placed together in the ward and survival outcomes are similar due to either shared physician treatment decision strategies or because patients have similar cancer types. For every spell, an admitting consultant physician is assigned—these physicians generally specialize in a given cancer type. Over the 9-year period of the study, there were a total of 73 admitting consultant physicians. However, only 24 of them saw at least 10 different patients. To retain model parsimony and avoid degeneracy, we included 24 indicator variables for these physicians. The referent group is therefore those patients who saw one of the 49 admitting physicians with less than 10 chemotherapy patients. For parsimony and space, we show only the physician with the largest positive and negative significant effects.

### 2.6 Analysis

To evaluate our hypotheses that social influence in a chemotherapy ward impacts patient mortality, we fit a series of Generalized Estimating Equations (GEEs) to account for the repeated measures of individuals with multiple chemotherapy cycles. We use a binomial variance with a logit link function to estimate the probability that an individual dies within 5 years of their last treatment. We fit several models using a step-wise blocked variable design. In our first block (Model 1), the outcome is modeled as a function of focal actor age, gender, time of chemotherapy cycle, number of treatment spells, the prognosis rank of cancer with which the patient was diagnosed, whether an individual had multiple tumor diagnoses (proxy for metastasis), total hours in the ward, and total person-hours of overlap with other patients. We then added the direct influence effects (Model 2) and then the indirect influence effects, or open two-paths (Model 3). Finally, as previously stated, patient sex may moderate the relationship between social contact (Shor & Roelfs, 2015). We, therefore, add interaction terms between the influence effects and whether the focal patient is male or female (Model 4).

We also conducted a number of robustness checks on this analysis and report the results in the Supplemental Information. Briefly, due to the issue previously discussed with the Jaccard-weighted person-hours, we dichotomize patient–patient overlap, calling significant any overlap that is more than expected by chance between two patients (*p*¡0.01) (Table S1 Model A, Figure S1). We also believe that a more parsimonious model could be created by treating cancer severity as a single continuous variable based on population-level cancer-specific death rates for patients in the United Kingdom. Thus, we report results using this alternative construction of cancer-severity in the supplement (Table S1 Model B). As well, we recognize that the GEE does not address latent confounding between connected individuals in a network, so we reran Model 2 (our main model) using bootstrapped samples of unconnected patients (Table S1 Model C). Because we have precise survival times, we fit a Cox proportional hazards model to our data (Table S1 Model D). Finally, although we have no data on the nurses assigned to patients, we conducted a sensitivity analysis in which we assume a patient’s primary nurse is a probabilistic function of both the nurses of their neighbors and their own health outcomes (and indirectly the health outcomes of their neighbors) (Figure S3).

## 3 Results

Table 1 reports the descriptive statistics of the sample. The 4,691 chemotherapy patients from Jan 1, 2000 to Jan 1, 2009 had a mean age of 59.8 (SD = 13.1), and 44% (*n* = 2,094) were male. The patients underwent a total of 43,898 chemotherapy spells, which consisted of an average of 8.5 (SD = 10.9) visits to the chemotherapy ward per spell with each visit lasting, on average, 4.0 hours (SD = 5.3). Two-thirds of the patients had a single diagnosis of cancer (*n* = 3*,* 122), 994 had two diagnoses, and one patient had nine diagnoses. With respect to location of the cancer, 1,108 patients were diagnosed with breast cancer—the most common cancer diagnosis—treated in the chemotherapy ward with a 5-year survival of 91% (based on UK statistics). Almost 500 patients were diagnosed with lung cancer; lung cancer is the second most severe cancer type with a 5-year survival of only 18% in the United Kingdom. A total of 850 people had a diagnosis of unspecified or multiple cancers that could not be classified to a single location. Finally, the 145 patients who were left-censored and therefore had no recorded cancer diagnoses had imputed values between 1.21 and 1.54 cancer diagnoses.

The mean total Jaccard index for neighbors’ survival was 6.50 (SD = 7.75) (Table 2, JI*_S_*_1_*_P_* ). The mean total Jaccard index for neighbors’ death was 10.67 (SD = 11.35) (JI*_S_*_1_*_D_*). With respect to 2-paths for neighbors’ survival or neighbors’ death, the means were 238.52 (SD = 243.60) and 385.01 (SD = 385.01), respectively (JI*_S_*_2_*_P_* and JI*_S_*_2_*_P_* ). The mean total Jaccard-weighted person-hours for neighbors’ survival was 698 (SD = 2*,* 237) (JW*_S_*_1_*_P_* ). The mean total Jaccard-weighted person-hours for neighbors’ death was 1,118 (SD = 3*,* 378) (JW*_D_*_1_*_P_* ). With respect to 2-paths for neighbors’ survival or neighbors’ death, the means were 21,027 (SD = 61*,* 611) and 34,658 (SD = 103*,* 810), respectively (JW*_S_*_2_*_P_* and JW*_D_*_2_*_P_* ). Thus, patients were more likely to experience deaths of co-present alters than survivorship; a similar pattern was observed for indirect ties through two-paths.

Results based on the GEEs using Jaccard-weighted person-hours are presented in Table 2. Being older, male, or having more severe cancer was associated with increased likelihood of death across all models (Model 1). This is consistent with previous literature and trends in cancer survival. The number of ward visits during a chemotherapy cycle and the total time of the cycle were not significant predictors of death. Additionally, the later in the study period a person began chemotherapy, the better their chance of survival, indicating a trend toward better treatment over time.

In Model 2, the 1-path influence parameters show a beneficial effect of neighboring patients having survived for 5-years (−0.346 CI: −0.540, −0.152) (JW*_S_*_1_*_P_* ), and an adverse effect of neighboring patients having died within 5-years (0.359 CI: 0.200, 0.508) (JW*_D_*_1_*_P_* ). Thus, there appears to be both positive and negative direct influence based on the health status of those one spends time with. Since these are per 1,000 Jaccard-weighted person-hours, and patients had an average of 698 Jaccard-weighted person-hours with patients surviving at least 5 years, patients will have the log-likelihood of mortality modified by an average of −0.232. Patients experienced an average of 1,119 Jaccard-weighted person-hours of overlap with patients surviving less than 5 years, the average patient’s log-likelihood of mortality increased by 0.401 based on this direct effect. When considering 2-path influence, the effects of non-transitive two paths to patients surviving at least 5 years survival is not significant (JW*_S_*_2_*_P_* ), but the result for non-transitive two paths with patients dying within 5 years was significant in Model 3 (−0.011 CI: −0.021, −0.00007) (JW*_D_*_2_*_P_* ). This latter result indicates some evidence of influence through open 2-paths. Finally, there is no significant moderating effect of sex (Model 4).

**Fig. 2:**
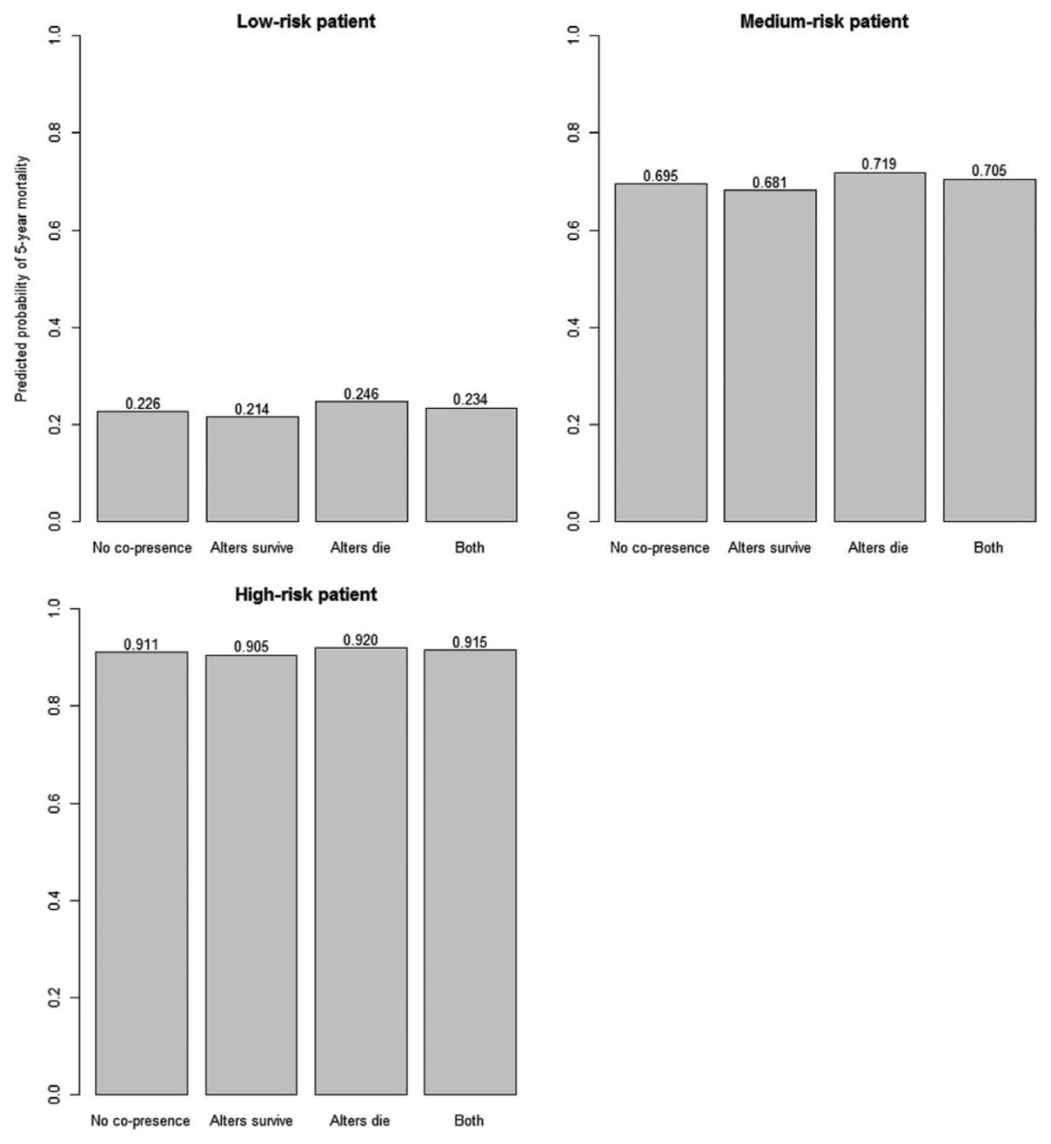
Predicted probability of 5-year mortality for patients with varying risk profiles and potential for social influence. Across panels, the first bar represents the predicted probability from model 4 with 0 for all influence terms. The average patient was one who had the median values for all covariates (rounded for dichotomous and categorical variables). This equates to a 69-year-old female whose chemotherapy lasted nine visits over 3 months and spent 30 hours in the ward starting in 2005, with a single diagnosis of a tumor of the ovaries. The low-risk and high-risk patients had values based on the first and third-quartile of the covariates depending on whether the relationship between 5-year mortality and the covariate was negative or positive, respectively. The low-risk patient was a 61-year-old female who visited the ward nine times over the course of a month and spent 30 hours in the ward starting in 2007, with a single tumor of the breast. The high-risk patient was a 79 year-old male whose chemotherapy included two visits to the ward over 4 months and spent 30 hours in the ward starting in 2003, whose primary diagnosis was cancer of the stomach, but had multiple cancer diagnoses. It is important to stress that these patients are not necessarily observed in these exact combinations of covariates; they are chosen in the way they were to demonstrate heterogeneity of the predicted probability of survival. Within each panel, influence terms were given the rounded mean value for the variable in question (refer to Table 2). No influence means the patient was co-present with no-one (never actually observed but gives a baseline probability). “Alters survive” means a patient was only co-present with patients surviving at least 5 years, and “alters die” means a patient was only co-present with patients dying within 5 years. “Both” means a patient was co-present with both types of patients.

To illustrate the influence of co-presence in the chemotherapy ward, we present the predicted probabilities from Model 2, for three hypothetical patients at varying levels of risk (Figure 2). The low, medium, and high-risk patients had predicted probabilities of death of approximately 23%, 69%, and 91%, respectively. For low-and medium-risk patients, we observe approximately a 2% change in predicted survival when comparing patients with no co-presence with those co-present with only patients having one type of outcome (survival or death). However, when patients are co-present with patients who die *and* patients who survive, their effect, on average, results in a minor decrease in predicted probability of survival for the patient. For the high-risk patient, smaller changes in predicted probability are observed due to the high baseline risk of death.

Our robustness analyses are, on average, consistent with our main findings. There are a few exceptions (Table S2 and Figure S2). Notably, the direct effect of co-presence with patients surviving 5 years is not significant when using an outcome of survival time instead of dichotomous survival. However, this analysis does not adequately adjust for intra-patient correlations. Additionally, the sensitivity analysis for nurse heterogeneity parallels the reduced robustness of the direct effect of co-presence with patients who survive at least 5 years evident from the survival analysis, assuming our simulation model adequately captures nurse-induced heterogeneity.

## 4 Discussion

In the current paper, we investigated whether cancer patient survival is associated with the survival of those with whom they are co-present during chemotherapy treatment. Our results suggest that co-presence matters. We find that a connected patient’s death increases the likelihood of the focal actor dying, and a connected patient’s survival decreases the focal actor’s chance of death. These results are approximately symmetric; being connected to a single survivor is similarly protective as being connected to a single non-survivor is deleterious to patient survival. There are no significant results for interactions between Jaccard-weighted person-hours and patient’s sex, which is not what we expected given previous findings (Shor & Roelfs, 2015). However, our study takes place in a previously unexplored social environment, so different mechanisms may be at play. Placing the results of this study in the context of cancer treatment, the magnitude of these results (Figure 2) is less than that of chemotherapy clinical trials but still clinically meaningful. Here, we observe a survival differential of 2% for patients at low to moderate risk when comparing no co-presence vs only co-presence with surviving patients, whereas effective chemotherapy clinical trials report survival differences of around 8% (Grabenbauer et al., 1993; Rapp et al., 1988). In effect, chemotherapy patient survival may be modified by one-quarter the quantity of the effect of the choice of chemotherapy.

Regarding the 2-path variables, we observe a non-significant result for the Jaccard-weighted person-hours to surviving patients two-steps away. At the same time, we found a significant effect of the Jaccard-weighted person-hours to patients dying within 5 years two-steps away, albeit marginally. As such, it would, therefore, only take a minimal amount of latent confounding between patients to remove that effect while leaving the main effect significant. Given the small magnitude of this effect and its significance level, even a small amount of measurement error could result in this finding. Therefore, we believe that the 2-path influence variables reinforce our main findings. However, future research should aim to more thoroughly test if this, and other network effects not measured here are in fact at play.

Based on the above results, the mechanism by which social influence occurs becomes clearer. We observe no significant association between total person-hours of overlap and one’s outcome, indicating that solely being around more people for more time on its own does not affect one’s health. In this context, just being around others receiving treatment with similar stressors does not seem to impart any health effects suggesting that social facilitation and social support are not the underlying influence mechanism. Previous research suggests that only 2/3rds of chemotherapy patients in the United Kingdom indicated receiving adequate emotional support from hospital staff (NHS, 2014). Thus, many patients may be actively seeking but not receiving support from others, particularly other patients, during treatment. Future research should therefore focus on whether and how support networks emerge in chemotherapy wards to elucidate the content of interactions we have detected via co-presence here. The influence effects between connected patients, on the other hand, likely reflect mechanisms where one’s outcome is related to the outcome of others, where either co-present patients form social relationships, or they observe others’ health trajectories. The mere observation of other cancer patients’ health changes over time may influence the observer’s own stress regarding their own cancer prognosis and subsequent health (Croyle & Hunt, 1991). This process is akin to social modeling; however, one may not consciously be altering how they respond to treatment. Rather, patients may see others doing better or worse, which might decrease or increase their stress, respectively, which in turn can impact their health.

Although network disruption was possible, it is unlikely that it is the mechanism given our results. If patients being removed from chemotherapy caused disruption to other patients’ networks in the ward, then we would expect to see adverse effects of focal actors’ neighbors finishing chemotherapy regardless of their neighbors’ outcomes. Instead we observe connected patient’ outcomes are positively correlated, indicating network disruption is not the mechanism underlying our findings.

Since the data used herein are observational, our results may stem from unmeasured confounding. We address the two forms we believe have the greatest potential to explain our results, but this is not an exhaustive list. First, patients may know one another prior to entering the ward, and any “social influence” observed here is the result of social interaction and influence outside of the chemotherapy ward due to preexisting social ties. If true, we detect social influence outside the chemotherapy ward via the measure of co-presence and in the presence of large amounts of noise. However, as we show in the Supplementary Information, we believe that the number of ties due to pre-existing relationships is likely minimal, limiting the effect this could have on our results. Second, nurse heterogeneity may explain our results if related both to patient outcomes and co-presence between patients. All of the nurses in the ward are specially trained as chemotherapy nurses, which would ideally limit heterogeneity across nurses (National Health Service, 2000). Furthermore, given our sensitivity analysis assuming nurse effects are on the same order of magnitude as physician effects (SI), we observe that our results are robust to nurse heterogeneity. Alternatively, if nursing effects (due to either nurse heterogeneity or other endogenous factors such as understaffing) do in fact explain our results, then we have detected meaningful nurse effects on patient survival previously not reported in the literature. Such potential effects should be investigated in the future.

With the above limitations in mind, this research has potentially important implications for the study of social networks. Whatever the underlying mechanism, we detect influence effects based solely on co-presence data from administrative records, which is much more efficient to gather than detailed relationship data. Although one may ask whether the ties used here really represent meaningful social ties, a recent survey found that 78% of patients in Britain said they would prefer to be treated in a communal setting indicating that patients feel they benefit from in close proximity to other patients (NHS, 2013). Thus, consistent co-presence represents the opportunity for patients to develop meaningful social ties during treatment. While it is unknown whether such ties developed amongst the patients studied here, it is clear that there is evidence of social influence among those who are consistently co-present. Moreover, we have employed a variety of novel approaches in our effort to rule out alternative explanations of our main findings. Future researchers may find our use of rank-order cancer diagnosis, cancer severity, physician effects, and holistic robustness checks valuable in their own work.

Our results imply that co-presence relates to health in the context of the chemotherapy ward. However, given the observed negative effects and possibility of unmeasured confounding, implementing changes to the ward and patient scheduling to benefit from this knowledge is difficult. If the observed social influence operates via changes to stress, then reducing patient stress in the ward without changing patient scheduling may be able to positively impact all patients. Oncologists can consider whether social influence may be at play in the wards to which they admit patients for chemotherapy, and whether scheduling to maximize co-presence of patients would *sui generis* be therapeutic. Given evidence that cancer support groups improve survival, patients could also be encouraged to engage in social support while in the chemotherapy ward, which could reduce stress (Cobb, 1976; Maria et al., 2004). Altering chemotherapy in this way can mitigate the deleterious effects of co-presence with patients experiencing negative outcomes and strengthen the positive effects of co-presence with surviving patients.

With these future directions and applications in mind, the findings in this paper are an important first step in describing the possibility of social influence occurring among patients co-present in a chemotherapy ward; a setting primarily for biological treatment, not treatment through social support and influence processes. Importantly, because we focus on mere co-presence, any findings not due to unmeasured confounding are likely to under-estimate the effect of social forces on health outcomes as co-presence represents the minimally necessary condition for influence. We hypothesize that the mechanism of this influence is mediated by stress response to co-presence with familiar others. Future research should focus on measurement of individual coping and stress processes in these settings to test this hypothesis directly.

## Supplementary Material

Supplementary info

## Figures and Tables

**Table 1:** Demographic characteristics of the 4,691 patients receiving chemotherapy at any time from Jan 1, 2000 to Jan 1, 2009.

Variable	Mean (SD) or *n* (%)
Age	59.79 (13.00)
Male	2094 (44%)
Number of ward visits during cycle	8.51 (10.94)
Time of chemotherapy cycle (years)	0.32 (0.48)
Average time in ward per spell (hours)	3.95 (5.32)
Number of cancer diagnoses	1.30 (0.63)
Primary cancer diagnosis	
Breast	1108 (24%)
Lung	443 (9%)
Pancreas	125 (3%)
Unspecified	850 (18%)
Other	2165 (46%)
Diagnosed with unspecified cancer	297 (6%)
Number of patients co-present with	113.91 (122.18)
Total person-hours of overlap	1012.66 (1,997,599)
JI*_S_*_1_*_P_* *	6.50 (7.75)
JI*_D_*_1_*_P_* *	10.67 (11.35)
JI*_S_*_2_*_P_* *	238.52 (243.60)
JI*_D_*_2_*_P_* *	385.01 (381.07)
JW*_S_*_1_*_P_*	697.94 (2,237.04)
JW*_D_*_1_*_P_*	1,118.61 (3,378.28)
JW*_S_*_2_*_P_*	23,027.58 (61,611.72)
JW*_D_*_2_*_P_*	34,657.70 (103,809.80)

* The JI terms are the JW terms divided by the person-hours of chemotherapy of the focal patient. In other words, they are the sum of the respective 1- or 2-path Jaccard indices for each focal actor.

**Table 2:** Results of generalized estimating equations modeling influence via Jaccard index. The model outcome is death within 5 years of ending chemotherapy. We used a binomial variance with logistic link function, and an unstructured covariance matrix for repeated outcomes on individuals.

	Model 1	Model 2	Model 3	Model 4
	Estimate (95% CI)	Estimate (95% CI)	Estimate (95% CI)	Estimate (95% CI)
Variable	Residuals^2^ = 871.7	Residuals^2^ = 859.6	Residuals^2^ = 857.6	Residuals^2^ = 854.8
Intercept	1.166 (0.166, 2.167)	1.257 (0.251, 2.263)	1.28 (0.275, 2.286)	1.266 (0.259, 2.272)
Age (years)	0.038 (0.032, 0.044)	0.038 (0.032, 0.044)	0.038 (0.032, 0.044)	0.038 (0.032, 0.044)
Sex (male)	0.216 (0.029, 0.404)	0.22 (0.032, 0.408)	0.219 (0.031, 0.408)	0.28 (0.083, 0.478)
Time of cycle (years)	−0.316 (−0.561, −0.071)	−0.351 (−0.608, −0.095)	−0.315 (−0.579, −0.051)	−0.359 (−0.626, −0.092)
Number of visits in course	0.003 (−0.009, 0.015)	−0.006 (−0.019, 0.007)	−0.01 (−0.024, 0.005)	−0.009 (−0.023, 0.006)
Years after 2000 patient begins chemotherapy	−0.123 (−0.16, −0.086)	−0.122 (−0.159, −0.084)	−0.121 (−0.159, −0.083)	−0.121 (−0.159, −0.083)
Total person-hours of overlap	0.01 (−0.001, 0.001)	0.001 (−0.001, 0.001)	0.001 (−0.001, 0.001)	0.001 (−0.001, 0.001)
More than one cancer diagnosis	1.177 (0.528, 1.826)	1.19 (0.535, 1.845)	1.197 (0.542, 1.851)	1.192 (0.536, 1.848)
JW*_S_*_1_*_P_* (per 1,000 person-hours)		−0.346 (−0.540, −0.152)	−0.282 (−0.536, −0.027)	−0.336 (−0.615, −0.057)
JW*_D_*_1_*_P_* (per 1,000 person-hours)		0.359 (0.200, 0.508)	0.457 (0.263, 0.651)	0.542 (0.317, 0.767)
JW*_S_*_2_*_P_* (per 1,000 person-hours)			0.011 (−0.002, 0.024)	0.117 (−0.002, 0.025)
JW*_D_*_2_*_P_* (per 1,000 person-hours)			−0.011 (−0.021, −0.00007)	−0.012 (−0.023, −0.001)
JW*_S_*_1_*_P_* X Sex(male) (per 1,000 person-hours)				0.168 (−0.259, 0.595)
JW_1_*_P_* X Sex(male) (per 1,000 person-hours)				−0.184 (−0.478, 0.109)
Has most severe cancer (Brain)*	1.07 (−0.012, 2.151)	1.028 (−0.054, 2.109)	1.008 (−0.074, 2.09)	1.015 (−0.067, 2.098)
Has least severe cancer (Prostate) *	−3.965 (−5.133, −2.797)	−4.012 (−5.181, −2.843)	−4.015 (−5.184, −2.846)	−4.057 (−5.227, −2.887)
Seen by oncologist with best average outcomes†	−2.025 (−4.344, 0.293)	−2.093 (−4.41, 0.224)	−2.09 (−4.41, 0.23)	−2.121 (−4.448, 0.206)
Seen by oncologist with worst average outcomes†	0.913 (0.074, 1.752)	0.903 (0.062, 1.744)	0.919 (0.076, 1.761)	0.918 (0.076, 1.76)

* Also adjusted for 18 other primary cancer types, including unspecified as one type.

† Also adjusted for 22 other physicians with at least 10 spells as the admission consultant.
