# Supplementary material for: Social influence on 5-year survival in a longitudinal chemotherapy ward co-presence network
Source: Netw Sci (Camb Univ Press). Author manuscript; Available in PMC 2018 Mar 1. (PMC5831372; doi:10.1017/nws.2017.16)
Supplement: Supplementary info [file NIHMS940456-supplement-Supplementary_info.pdf]

Supplemental Information

Consistent co-presence measure

Although the Jaccard index provides a continuous, undirected measure that is both straightforward to compute and whose properties are well-known, it results in very high values for individuals who are only in the ward for short periods of time, hence our creation of the Jaccard-weighted person-hours of overlap. However, we observe significant heaping at certain values of the Jaccard index (Figure S1). This heaping likely represents underlying hospital policies that result in patterns of patient overlap which are not controlled for in the Jaccard-weighted person-hours. Moreover, the Jaccard-weighted person-hours makes the assumption that social influence can occur after a moment of co-presence, which we believe is somewhat unrealistic since previous work has shown that the likelihood of influence is positively correlated with the strength of a relationship (Aral & Walker, 2014). We therefore consider a second measure of co-presence that defines patients as connected when they are consistently co-present in the ward. This allows us to check the robustness of, and supplement our primary analysis.

We posit that two patients are consistently co-present if their chemotherapy treatments overlap more often than would be expected by chance. To derive this variable, we first define a meaningful cutoff in terms of time co-present. As previously stated, a patient's chemotherapy visits are primarily determined by their first visit and the standard schedule for their prescription. We assume a patient's first visit could vary plus or minus one day based on bed availability and patient preference. For example, assume a patient's first treatment spell is on March 15. The window for overlap is therefore March 14-16. All patients who had a spell within this window represent the corresponding risk set of patients with whom the focal patient could have overlapped. The window defining the risk set excluded weekends; thus, the risk set window for

a patient scheduled to receive treatment on a Monday included the previous Friday and the following Tuesday. Based on these assumptions, we determine how often each patient would have overlapped with others conditional on when chemotherapy began. We observe with whom overlap and for how long they overlap based on a random sample from the risk set, assuming the periodicity of chemotherapy holds. By repeating this procedure 1000 times for each patient, we create a patient-specific empirical distribution of overlap times, and draw a cutoff at the 99th percentile of this distribution, forming an edge between the focal actor and the other patient. Formally, this can be written as:

$$A_{ij} = \begin{cases} 1 \; if \; |H(i) \cap H(j)| > Q_{99}(|H(i) \cap H(k)|) \;\; \forall \; k \\ 0 \; otherwise \end{cases}$$

Where *H(i)* is the set of hours spent in the ward by patient *i* and Q_{99} is the 99th percentile of that patient's co-presence times with other patients based on the empirical distribution. For a visual explanation of this method, see Figure S2.

Thus, an edge in this co-presence network is drawn between two patients when the amount of time spent co-present in the ward is greater than the time at least one of the patients in question spends with 99% of the risk set of patients randomly sampled. We will refer to the edges in this network as an indicator of patients who are "consistently co-present". Unlike the Jaccard-weighted person-hours, A_{ij}=0 does not imply that there was 0 overlap between *i* and *j*, only that we do not consider such overlap significant, that is, *i* and *j* are not consistently co-present. While this method results in a directed network (patient *i*'s overlap with patient *j* may be significant for patient *i* but not patient *j*), nearly all ties were mutual (>99%). Given that empirically nearly all ties are mutual and theoretically co-presence is symmetric, we treat the network as undirected. Thus, edges in the network have the convenient interpretation that two

patients are connected when at least one of them was consistently co-present with the other. The resulting network is shown in Figure S3.

Although the method to determine this network is stochastic, over 99% of the ties were the same between runs, we create the network one time using 1000 samples of patient schedules. Using this network, we construct counts, for each focal patient, of the number of connected or 2-path patients who survive 5 years following their chemotherapy and the number of connected or 2-path patients who die within 5 years following their chemotherapy at the time the focal patient finishes their chemotherapy. We then fit a GEE with these predictors and the same covariates as in the main model (Table S1, Model A). Due to the dichotomous nature of the network, we also observe a number of isolates (~50% of patient), so we also include an indicator of whether the focal patient was an isolate or not in order to distinguish between those who have small amounts of overlap with many patients vs those who have a lot of overlap with few patients.

In the consistent co-presence network GEE we fit a term for whether an individual had *any* significant co-presence (all individuals had at least one weighted edge in the Jaccard index network). The term for whether an individual is an isolate indicates that isolates were more likely to die within five years than those co-present with other patients (0.361 CI:0.195,0.526). Thus, patients benefit from significant co-presence with at least one other patient in the ward, irrespective of alters' outcomes.

We observe similar effects as those observed when using Jaccard-weighted person-hours for both a significant positive effect between 5-year mortality and the number of consistently co-present patients who died within 5 years following their chemotherapy (0.103 95% CI: 0.064,0.142), and a negative significant effect between 5-year mortality and the number of

consistently co-present patients who survived for at least 5 years following their chemotherapy (-0.117 95% CI: -0.175,-0.059).

These results indicate that our primary analysis using Jaccard-weighted person-hours is not unduly affected by allowing for co-presence of short periods of time to impact patient health. However, the coefficient of the “isolate” variable indicates there may be some sort of threshold effect of consistently spending time with the same person or people. Although the model was stochastic, 99% of individuals stayed the same with respect to “isolate” status across repeated simulations. In all models we include a variable for the total person-hours of co-presence, which is not significant. We separately included a variable for the number of people with whom one was co-present, which was also not significant (we did not include it in the reported models because including both it and the person-hours of overlap caused the model to not converge). This indicates that it is likely not diversity of partners with whom one is co-present that impacts health, but consistent co-presence with the same individuals. However, the exact threshold used here is likely not perfect; future work should examine if this is the case, and if so, where such a threshold in consistent co-presence lies.

Cancer severity as a continuous variable

Although we treat cancer severity as a series of dummy variables for the primary cancer diagnosis, we recognize this might not be the most parsimonious way to do so. Additionally, this method averages out the correlation with mortality across the study period. If the prognosis changes (as we observe a significant decrease in mortality the later during the study period one begins chemotherapy), the method we use will not account for this. However, if we treat the cancer type as a rank-ordered variable based on the predicted-year mortality, we save degrees of

freedom in the model. Additionally, although the prognoses of the types of cancer may change during the study period, the rank-order should be less susceptible to change. We therefore present the results of modeling cancer severity as a series of dummy variables here (Table S1, Model B). The results are parallel to the main results measure in that both co-presence with surviving (-3.438 CI: -5.381,-1.494) and co-presence with dying patients (3.570 CI: 2.044,5.096) are significant.

Correlations between patients

It should be noted that the GEE does not adjust for the correlation *between* patients. Patients within two steps of each other may have correlated survival/death counts (e.g. if patients A and B are both connected to patient C, and C survives, A and B's count are correlated) and may also have other unmeasured latent factors which are correlated between them. To examine whether this had an effect on the model inferences, we reran the GEE with random samples of patients who are all at least two steps away from one another and should be largely independent. These models, run using 1000 samples, give results that are generally in the same direction and approximately the same magnitude as the models with all the data included (Table S1, Model C). Generally, the spread of coefficients is greater than the variance of the coefficients from the full model. Although this is not a rigorous test for how robust these results are, some of the variables seem to have extremely consistent correlation with the outcome (e.g. age, sex and cancer severity), whereas others are not (total hours in the ward and time of chemotherapy cycle). Importantly, the Jaccard-weighted person-hours of co-presence with patients dying within 5 years had a median value very similar to the point estimate from the full model, indicating that the finding for that variable was reasonably reliable. The median value for the Jaccard-weighted person-hours with patients surviving 5 years also had a median value very similar to the point

estimate from the full model. Additionally, the correlation between the total Jaccard-weighted person-hours for patients within one or two steps of one another is not significantly different from the correlation between the total Jaccard-weighted person-hours for patients more than two steps away from one another (-0.013 vs. -0.012, p=0.68), so we do not believe that correlation between patients near one another in the network is an issue.

Survival Analysis

Although our outcome of interest is 5-year survival, our data include precise patient survival times. As such, we also fitted a Cox proportional hazard model to evaluate the robustness of the inferences (Table S1, Model D) (Mills, 2011). This allows assessment of the hazard of death accounting for survival time instead of just the probability of death. We observe that the results are generally similar to our main results, except there is a non-significant relationship between the Jaccard-weighted person-hours with patients surviving for 5 years and patient survival. This may in part be due to the lack of control for intra-patient correlations as in the GEE.

Sensitivity Analysis for Nurses

We recognize that nurse heterogeneity could affect the health outcomes of patients and also be correlated to patient-patient co-presence, which could explain our results. We were unable to obtain data on nursing staff, but we present here a sensitivity analysis. First, we create *n* nurses, ranging from 5 to 95 and randomly assign each a quality of care parameter. This parameter takes a normal distribution with mean=0 and sd=0.65, the distribution of physician parameters from Table 2 Model B, meaning we assume that the effects of nurse heterogeneity are approximately the same magnitude and distribution as physician heterogeneity (which we

observe). Each patient is assigned a primary nurse either based on assortative mixing with the nurses of their neighbors or their own health outcome. The probability of whether the nurse is based on assortative mixing or patient outcome was chosen to range from 0.05 to 0.95. If the nurse is assigned based on the neighbor(s)' nurse(s) one nurse is chosen at random among neighbors set with probability proportional to the Jaccard index with those neighbors, which is written as:

$$P(N_i = N_j) \propto J_{ij}$$

Where N_i is the nurse assigned to patient i.

If the nurse is chosen based on the patient's outcome, then a nurse is chosen with probability proportional to the probability of survival if the patient survived, or proportional to the probability of death if the patient died. We assume the nurse heterogeneity parameter relates linearly to the log-odds of survival (because the physician parameters do), and so calculate the probability straightforwardly. For this probability, we assume all patients have the population mean probability of survival (33%) as their baseline which is modified only by the nurse heterogeneity parameter. This is written as:

$$P(N_A = n) \propto \begin{cases} \frac{\exp\left(\log(0.5)+HN(n)\right)}{1+\exp\left(\log(0.5)+HN(n)\right)} \; if\ S(A) = 1 \\ 1 - \frac{\exp\left(\log(0.5)+HN(n)\right)}{1+\exp\left(\log(0.5)+HN(n)\right)} \; if\ S(A) = 0 \end{cases}$$

Where HN(n) is the heterogeneity parameter for nurse n. This results in 95% of probabilities of survival ranging from 12.0% to 64.7%. Assuming the patient survived, a nurse with a heterogeneity parameter at the 97.5th percentile of the distribution would be chosen 5.4 times more often than a nurse with a heterogeneity parameter at the 2.5th percentile. This forced

association between nurse parameter and patient outcome is not meant to precisely reflect how nurses are assigned in the ward, but rather to fulfill the necessary condition that nurse assignment is correlated with outcome to induce confounding. Each combination of number of nurses and assortativity probability was run 100 times, and the proportion of significant ($p<0.05$) direct effects were recorded (Figure S3).

Overall, we see that the significance of the main findings is relatively robust to the number of nurses. We observe that the fraction of significance falls below 50% when the probability of assigning nurse based on adjacent patients' nurses falls between 0.55 and 0.65 for the effect of co-presence with patients surviving at least 5 years, and between 0.75 and 0.85 for the effect of co-presence with patients dying within 5 years. These results show that our results are relatively robust to nursing effects on the order of physician effect, since at any given time there are multiple nurses in the chemotherapy ward, and nurses generally take on patients as the nurses are available. This means that many patients will not have the same primary nurse as the patients with whom they are co-present, so the high values we observe are needed to remove our results are likely unrealistic.

That being said, we find that the effects of co-presence with patients surviving 5 years are less robust, as in our survival analysis. This may in part be due to the prevailing outcome of 5-year mortality and so less variability in co-presence times with surviving patients. It may also reflect real uncertainty in the result that we cannot eliminate with the present data. Additionally, this analysis assumes that, conditional on mortality status and co-presence, nurses are assigned randomly. However, patients' admission consultants are not related to covariates such as age or gender. We therefore believe that this assumption of random assignment reflects reality, particularly given the fact that nurses do not specialize beyond general chemotherapy.

Ties concurrent with pre-existing social ties

Given that our study population is drawn from a relatively small catchment area, it is possible that our belief that patients in the chemotherapy ward do not know one another prior to initiating chemotherapy is incorrect. However, we believe ties of this sort are very unlikely, as they would stem from the confluence of a number of unlikely events. First, both patients in a dyad would need to be diagnosed with cancer, the lifetime risk of which in the UK is ~50% (UK, 2014). Second, both patients would need to know one another. Given a population size at risk of cancer in Oxfordshire is 80,000 and that each person knows on average 600 individuals (McCormick, Salganik, & Zheng, 2010), each pair of individuals has an 0.75% chance of knowing one another under a random mixing model. Finally, the individuals must be diagnosed around the same time, have similar availability schedules, and go to the same clinic, to be on chemotherapy concurrently. Given that most cancers occur between the ages of 50 and 80, this occurs one time in thirty if we non-conservatively assume that a year qualifies as "around the same time". If we assume these events are independent then the probability of two patients knowing one another and being on chemotherapy around the same time is 0.0000625. Given that each patient sees 115 patients on average in chemotherapy for at least one hour, this only nets out to an average of about 0.007 such alters per patient stemming from pre-existing social ties. We therefore do not believe that such preexisting ties would be a large enough presence to affect our results.

Supplementary Figures and Tables

Figure S1. Kernel density-smoothed function of Jaccard indices. We observe heightened frequency of Jaccard index values at 1, $^{1}/_{2}$, $^{1}/_{3}$, $^{1}/_{4}$, and $^{1}/_{5}$, which indicates some sort of endogenous underlying process influencing patient ward spells and therefore overlap not accounted for by the Jaccard index.

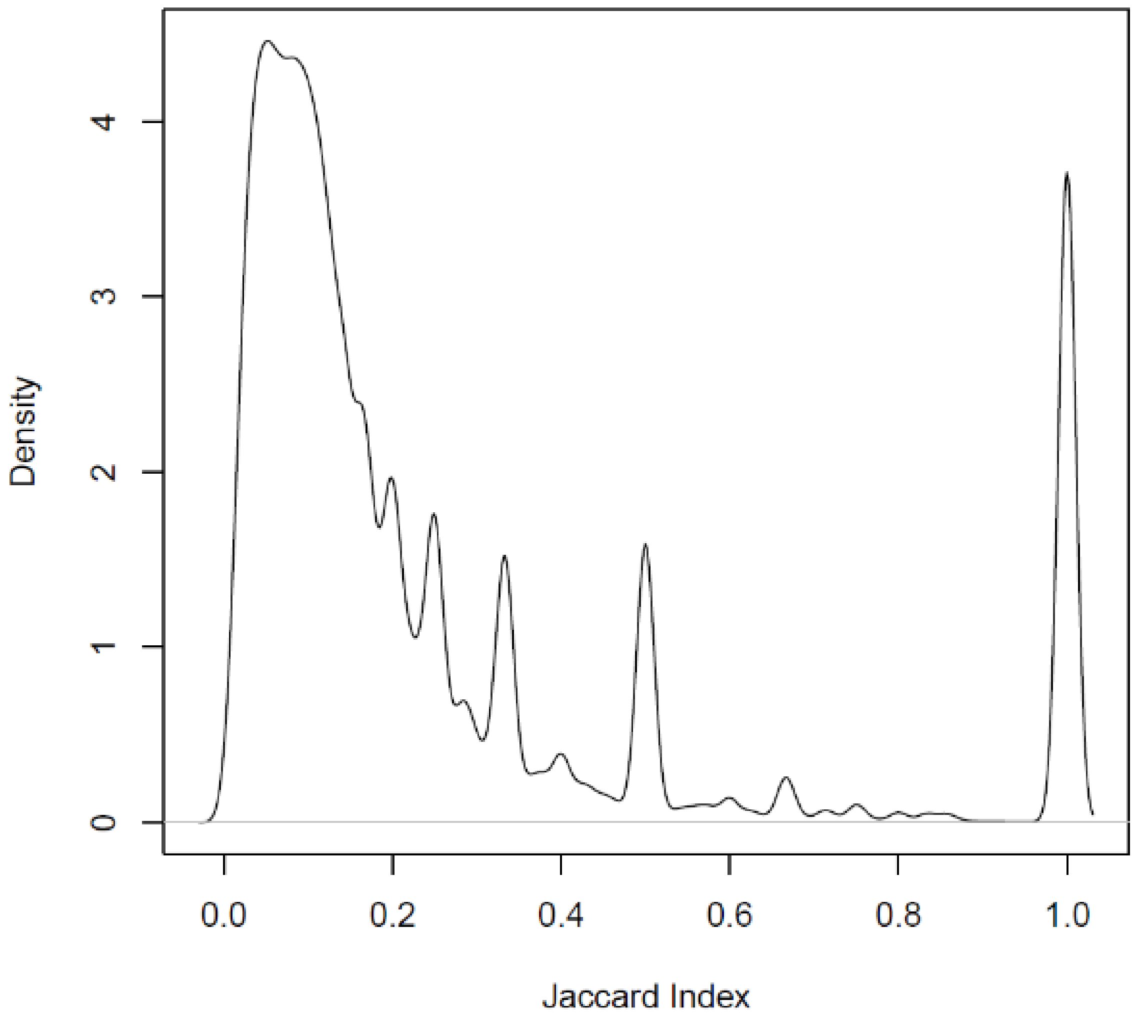

Figure S2. Heuristic vignette of what constitutes consistent co-presence. Colored blocks indicate the hours each of 4 patients are present in the ward over three different days. A) shows a case where patient A overlaps with patient B for all three spells. However, had A's first spell been 5 hours earlier or later, they would have overlapped with C or D, respectively just as much as they overlapped with B, so their overlap with B is not more than expected by chance due to random variation in the first spell, and therefore A and B are not consistently co-present. B) shows a case where A and B would be considered consistently co-present. Here, A still overlaps with B during all three spells. Had A's first spell been moved earlier or later, A would not have greatly overlapped with any other patients, so A's overlap with B is greater than that expected by chance. This therefore controls for the underlying scheduling possibilities not accounted for by the Jaccard-weighted person-hours.

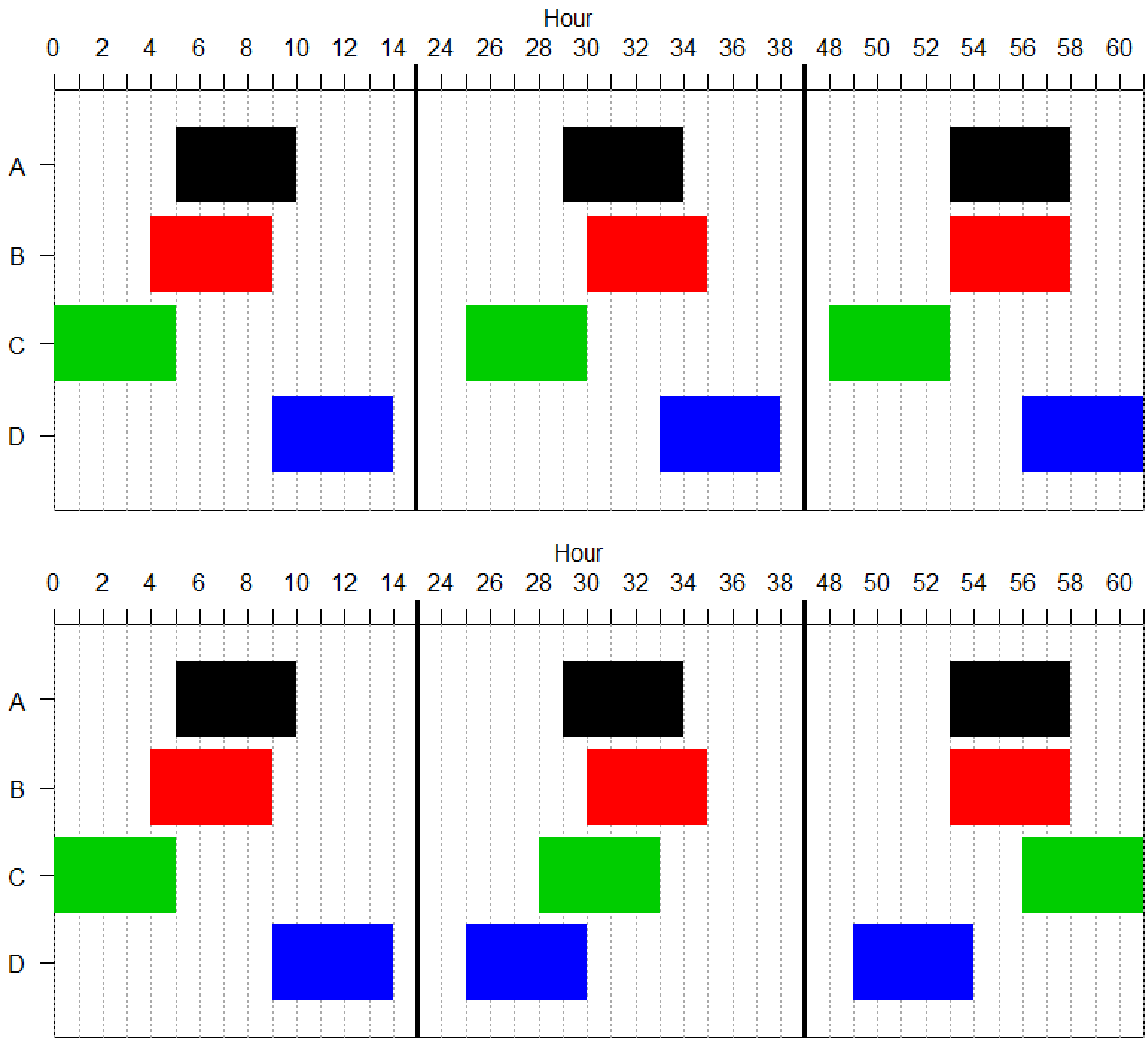

Figure S3. Exemplary largest connected component of network overlap among chemotherapy patients from 2000 to 2009 (n=2,228). An edge exists between two patients if they were co-present in the chemotherapy ward more than expected (p<0.01). Node color ranging from white to red indicates the week at which each patient began chemotherapy, representing the temporal nature of this network (with white values corresponding to January 1^{st,} 1998). The edge color value indicates the amount of time the two connected patients spent together in the ward (darker edges representing more time co-present in the ward).

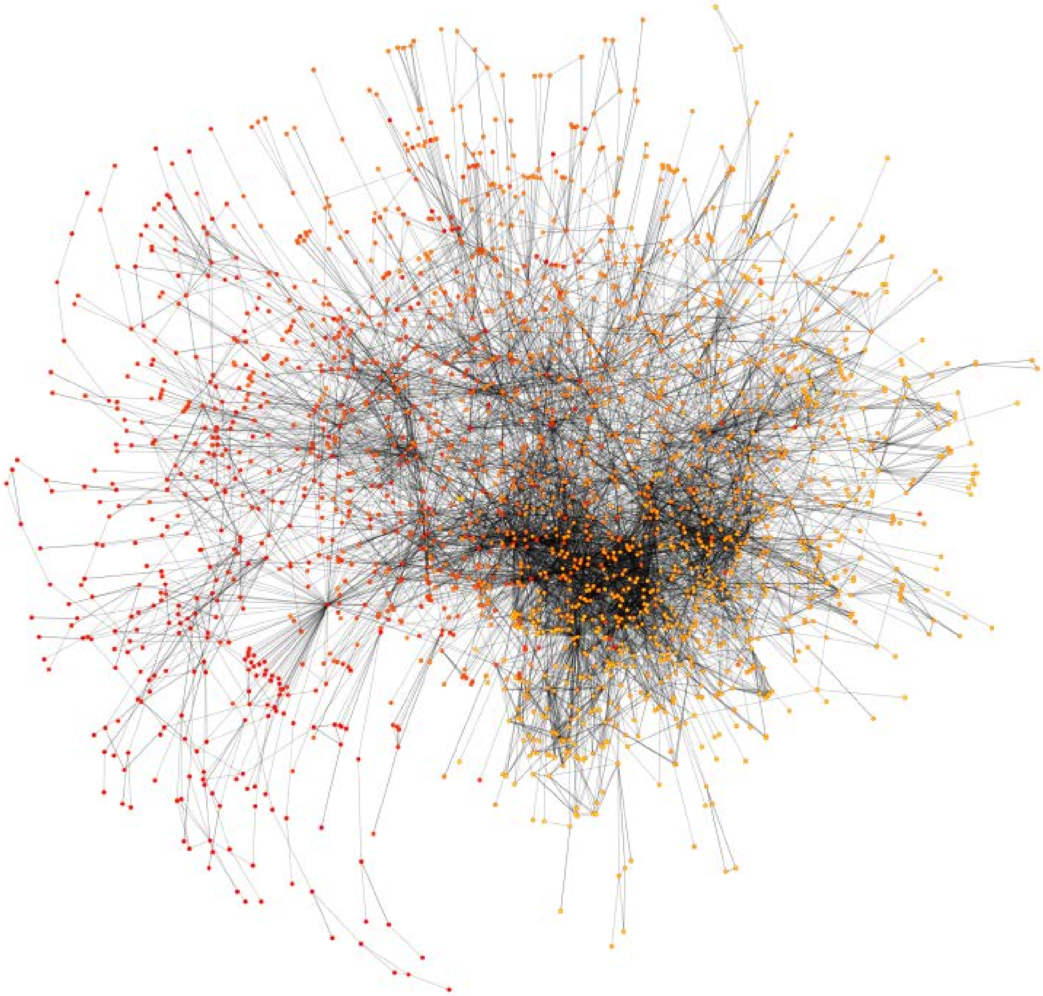

Table S1. Results of sensitivity analyses. The first 3 models are GEEs constructed in the same way as the primary analysis but with specific changes. A) Uses the consistent co-presence metric which is dichotomous, and also includes an indicator variable of whether an individual is an isolate or not. B) Treats cancer severity as a categorical variable. C) Is based on a sample of patients who were not co-present with one another to remove correlation between variables. Results are based on 100 trials of sampling a subset of patients in this way. D) Instead of a dichotomous 5-year survival outcome, we treat survival time as the outcome of interest, using a Cox proportional hazards model.

	**A) Consistent co-presence measure**	**B) Treat cancer severity rank as a continuous rank-ordered variable**	**C) Sample a subset of independent patients**	**D) Survival Analysis**
Variable	Estimate (95% CI)	Estimate (95% CI)	Median Estimate (Min,Max)	Log(Hazard Ratio) (95% CI)
Intercept	1.102 (0.482,1.722)	1.23 (0.613,1.847)	1.402 (-12.886,3.422)	N/A
Age (years)	0.041 (0.035,0.046)	0.04 (0.035,0.046)	0.045 (0.026,0.073)	0.022 (0.020,0.025)
Sex (male)	0.306 (0.133,0.479)	0.341 (0.168,0.514)	0.439 (-0.095,1.052)	0.215 (0.134,0.296)
Time of commencing chemotherapy (years after 2000)	-0.092 (-0.131,-0.053)	-0.216 (-0.498,0.067)	-0.201 (-0.381,-0.012)	-0.075 (-0.097,-0.054)
Time of cycle (years)	0.001 (-0.295,0.297)	-0.014 (-0.029,0)	-0.259 (-3.182,4.037)	-0.070 (-0.177,-0.037)
Number of visits in cycle	-0.004 (-0.019,0.012)	-0.154 (-0.195,-0.113)	-0.053 (-0.495,0.237)	-0.015 (-0.021,-0.009)
More than one cancer diagnosis	1.14 (0.561,1.716)	0.056 (-0.28,0.392)	1.740 (-0.117,16.344)	0.352 (0.310,0.394)
Has most severe cancer (Brain)	0.971 (-0.950,2.037)	1.077 (0.503,1.65)$^{\beta}$	1.061 (-2.009,3.13)	0.971 (-0.950,2.037)
Has least severe cancer (Testes)	-4.023 (-5.023,-3.023)	N/A	-4.143 (-6.232,-1.145)	-4.121 (-5.279,-2.963)
Total person-hours of overlap	0.001 (-0.001,0.001)	0.001 (-0.001,0.001)	0.001 (-0.002,0.004)	0.001 (-0.001,0.002)
Total hours in the ward	-0.001 (-0.006,0.006)	-0.001 (-0.006,0.006)	-0.001 (-0.155,0.115)	-0.001 (-0.002,0.001)
Physician with worst average outcomes$^{\alpha}$	1.047 (0.701,1.392)	1.067 (0.716,1.418)	1.132 (0.321,2.145)	0.371 (0.247,0.496)
Physician with best average outcomes$^{\alpha}$	-2.041 (-4.021,-0.062)	-1.916 (-3.866,0.034)	-2.055 (-2.889,1.054)	-1.90 (-3.864,0.0621)
Is patient an isolate?	0.361 (0.195,0.526)	N/A	N/A	N/A
JW_{S-1} (per 1,000 person-hours)	-0.117 (-0.175,-0.059)$^{\gamma}$	-0.344 (-0.538,-0.149)	-0.290 (-0.905,0.823)	-0.598 (-1.249,0.053)
JW_{D-1} (per 1,000 person-hours)	0.103 (0.064,0.142)$^{\gamma}$	0.357 (0.204,0.510)	0.851 (-0.301,1.143)	0.761 (3.256,1.197)

α: Also adjusted for 22 other physicians with at least 10 spells as the admission consultant

β: In model B, rank of cancer severity was modeled as a continuous variable instead of a series of dummy variables.

γ: For the consistent co-presence measure, the units are total number of paths

Figure S4. Heatmap represents results of sensitivity analysis for effects of nurse heterogeneity.

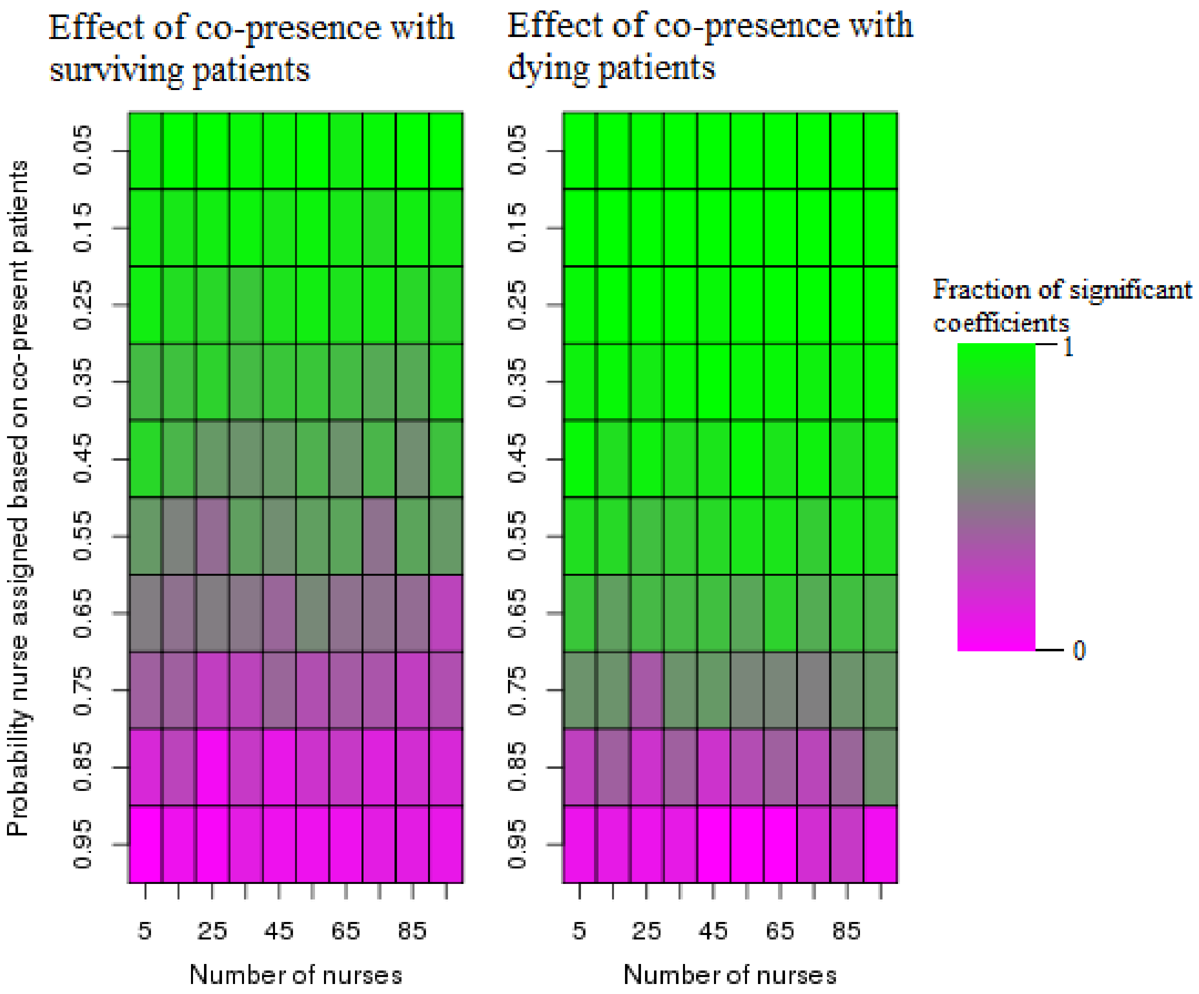

References

Aral, S., & Walker, D. (2014). Tie Strength , Embeddedness , and Social Influence : A Large-Scale Networked Experiment. *Management Science*, *6*, 1352–70.

McCormick, T. H., Salganik, M. J., & Zheng, T. (2010). How many people do you know?: Efficiently estimating personal network size. *Journal of the American Statistical Association*, *105*(489), 59–70. http://doi.org/10.1198/jasa.2009.ap08518

Mills, M. (2011). *Introducing Survival and Event History Analaysis*. London: SAGE Publications Ltd.

UK, C. R. (2014). Cancer Mortality Statistics. Retrieved from http://www.cancerresearchuk.org/health-professional/cancer-statistics/mortality?a=5441
